# Steel Strip Surface Defect Detection Method Based on Improved YOLOv5s

**DOI:** 10.3390/biomimetics9010028

**Published:** 2024-01-03

**Authors:** Jianbo Lu, Mingrui Zhu, Xiaoya Ma, Kunsheng Wu

**Affiliations:** 1 Guangxi Key Lab of Human-Machine Interaction and Intelligent Decision, Nanning Normal University, Nanning 530001, China; lujianbo@nnnu.edu.cn (J.L.); 418229223@email.nnnu.edu.cn (M.Z.); kujyeer@email.nnnu.edu.cn (K.W.); 2 Department of Logistics Management and Engineering, Nanning Normal University, Nanning 530023, China

**Keywords:** YOLOv5s, strip surface defect detection, CBAM, CSBL, SPPF-A

## Abstract

Steel strip is an important raw material for the engineering, automotive, shipbuilding, and aerospace industries. However, during the production process, the surface of the steel strip is prone to cracks, pitting, and other defects that affect its appearance and performance. It is important to use machine vision technology to detect defects on the surface of a steel strip in order to improve its quality. To address the difficulties in classifying the fine-grained features of strip steel surface images and to improve the defect detection rate, we propose an improved YOLOv5s model called YOLOv5s-FPD (Fine Particle Detection). The SPPF-A (Spatial Pyramid Pooling Fast-Advance) module was constructed to adjust the spatial pyramid structure, and the ASFF (Adaptively Spatial Feature Fusion) and CARAFE (Content-Aware ReAssembly of FEatures) modules were introduced to improve the feature extraction and fusion capabilities of strip images. The CSBL (Convolutional Separable Bottleneck) module was also constructed, and the DCNv2 (Deformable ConvNets v2) module was introduced to improve the model’s lightweight properties. The CBAM (Convolutional Block Attention Module) attention module is used to extract key and important information, further improving the model’s feature extraction capability. Experimental results on the NEU_DET (NEU surface defect database) dataset show that YOLOv5s-FPD improves the mAP50 accuracy by 2.6% before data enhancement and 1.8% after SSIE (steel strip image enhancement) data enhancement, compared to the YOLOv5s prototype. It also improves the detection accuracy of all six defects in the dataset. Experimental results on the VOC2007 public dataset demonstrate that YOLOv5s-FPD improves the mAP50 accuracy by 4.6% before data enhancement, compared to the YOLOv5s prototype. Overall, these results confirm the validity and usefulness of the proposed model.

## 1. Introduction

As a major product of the iron and steel industry, strip steel has been widely used in machinery manufacturing, automotive, shipbuilding, and aerospace industries. The quality of its surface directly affects the performance and quality of the final product [1]. However, due to the influence of raw materials, structure, manufacturing processes, and other factors, the surface of strip steel often exhibits defects such as cracks, scratches, inclusions, patches, pockmarks, and iron oxide. These defects can impact the performance and appearance of strip steel to varying degrees and may even result in significant economic losses. Therefore, employing computer image processing and other technologies to detect surface defects on strip steel is of great significance in improving its quality.

With the increasing production speed of strip steel, manual visual inspection in the early stages cannot meet the production demand. Infrared detection is limited by the limited absorptive capacity of infrared light, and the classification accuracy is not high. Laser scanning is more accurate than the former but requires a stricter factory environment as dust particles can affect the reflection of the laser light [2].

Li et al. [3] used a Symmetric Surround Saliency Map for surface defect detection and Deep Convolutional Neural Network (CNN) for strip steel defect classification using the NEU dataset. X. Feng et al. [4] used RepVGG (Making VGG-style ConvNets Great Again) in combination with SA (spatial attention) spatial attention to achieve good results in strip defect detection. I. Konovalenko et al. [5] used Resnet50 (Residual Neural Network) for strip defect detection with an accuracy of more than 90%. Compared with traditional detection methods, convolutional neural networks for image recognition offer the advantages of high detection efficiency and accuracy [6]. Currently, deep learning has gradually become the primary technique for strip surface defect detection due to its powerful modeling capability and efficient inference. Numerous studies have shown that images obtained by neural networks are of higher quality compared to those enhanced by traditional methods [7].

Q Ren et al. [8] applied Faster R-CNN (Towards Real-Time Object Detection with Region Proposal Networks) to strip defect detection, and M Tang et al. [9] fused Resnet with the proposed multi-scale maxpooling (MSMP). These algorithms belong to the category of two-stage target detection algorithms, which undergo two steps involving the RPN (Region Proposal Network) network and target classification and identification. This process is slow. On the other hand, single-stage detection algorithms such as YOLO (You Only Look Once) [10,11] and SSD (Single Shot Detector) [11] offer faster detection speed and efficient computational performance, making them more suitable for real-time image detection.

YOLOv5 has been optimized extensively in terms of image input. Adaptive image scaling improves compatibility with the dataset, and adaptive anchor frame calculation eliminates the need for running additional programs. Additionally, YOLOv5 divides the residual structure into two CSP structures, streamlining the computational effort while ensuring accuracy. Researchers have successfully applied the YOLO algorithm to steel defect detection and achieved good results. Zheng et al. [12] improved YOLOv3 by using MobileNet as the backbone network and added the HaloNet and SENet attention modules for better detection. Xie et al. [13] combined deep separable convolution with the YOLO network and achieved 83.9% accuracy on the NEU dataset. Zhao et al. [14] combined filtered weighted feature vectors with YOLOv5x and added a convolutional layer behind a spatial pyramid structure, resulting in an mAP value of 87.3% and a 5% improvement. M A et al. [15] used the improved YOLOv4 for surface defect detection on aluminum strips and achieved a 96.28% mAP using two-channel attention with an optimized metric. Xiao et al. [16] applied Bi-FPN in combination with YOLOv5 for the detection of defects on the surface of zinc bright flakes on galvanized steel, and obtained good results. The classical YOLOv5 will be difficult to converge the model when dealing with strip steel production images due to the special of strip steel images, while for YOLOv7, although the model structure and training effect have been improved, there is not much difference in the detection speed and detection accuracy, and its parameter count is large and very dependent on the pre-training. In addition, because the strip steel pictures are taken on the factory floor, the imaging light and background have a great influence, and the defect features are characterized by small chromatic aberration, dense distribution and slenderness, which are difficult to classify using the standard pre-training model [17].

Therefore, it is necessary to develop stable, reliable, and robust detection methods for fine-grained features that can adapt to light changes, noise, and other external adverse environments. Additionally, collecting a large number of defect samples and maintaining category balance in the industrial field is challenging. This limitation can reduce the detection performance and robustness of deep learning methods that require a substantial amount of training data.

In this paper, YOLOv5s-FPD is proposed as an improvement over the YOLOv5 model. YOLOv5s-FPD adjusts the structure of SPPF (Spatial Pyramid Pooling Fusion) by placing more emphasis on the global and detailed features of the strip steel. This adjustment allows for better identification of defective features. Additionally, YOLOv5s-FPD balances detection accuracy and speed by constructing the CSBL (Channel and Spatial-wise Bottleneck Layer) multi-scale feature module, which reduces model size and speeds up processing. The introduction of ASFF (Adaptive Spatial Feature Fusion) and CARAFE (Content-Aware ReAssembly of FEatures) enhances the sensitivity of the receptive field to fine-grained features, resulting in improved performance of the spatial pyramid structure. Moreover, to facilitate practical application in strip defect detection, this study proposes a convenient data enhancement method that allows for easy adjustment of type and size.

In summary, the main innovations of this study are as follows:

1. The proposed YOLOv5s-FPD model demonstrates significant superiority. It achieves a 2.6% improvement in accuracy before data enhancement and 1.8% improvement after data enhancement. It achieves recognition accuracy for all six defects in the dataset, reaching the top level. Furthermore, on the VOC2007 public dataset, YOLOv5s-FPD achieves a 4.6% improvement in mAP50 accuracy compared to the prototype YOLOv5s.
2. By proposing the SPPF_A fine-grained target detection framework, this study strengthens the weights of small targets in the spatial pyramid. This addresses the issue of poor detection for small and dense targets, ultimately improving the model’s accuracy in defect detection of picture features.
3. The CSBL multi-scale feature module is constructed in this study, which combines the spatial pyramid pattern with the simple separable convolution 2SConv. This reduces the number of convolutional parameters while maintaining the effectiveness of convolution. Additionally, it enables deeper hierarchical information mining, resulting in improved detection results.

The rest of the article is structured as follows: Section 2 presents a literature review, including a comparison of strip defect object detection models, strip defect datasets, and the YOLO method. Section 3 focuses on the Improved YOLOv5s-FPD algorithm, describing specific architectural details of the improved algorithm’s backbone network. Section 4 presents an analysis of the data experiments, followed by Section 5 for discussion, and Section 6 for the conclusion.

## 2. Related Work

### 2.1. Strip Defect Detection Technology

Among recent models, the two-stage network architecture of Faster R-CNN (Fast Region-based Convolutional Network) has shown better results when dealing with high accuracy, multi-scale, and small objects [18]. Wei et al. [19] introduced weighted RoI (Region of Interest) pooling to reduce region misalignment caused by Faster R-CNN quantization, improving the mean average precision (mAP) of surface defect detection from 97%. However, this improvement may impact the processing speed. Wang et al. [20] combined the improved Faster R-CNN with a homemade dataset for accurate surface wear recognition. Faster R-CNN as a two-order network architecture can more accurately solve multi-scale and small target problems in surface defect detection. In addition, Faster R-CNN is convenient for migration learning and robust. However, because Faster R-CNN uses a fully connected layer at the end, the problem of long network computation time has not been solved. In contrast, the SSD (Single Shot MultiBox Detector) algorithm opts for a one-stage network architecture and proposes a similar concept of anchor boxes as in Faster R-CNN. SSD demonstrates a clear speed advantage over Faster R-CNN without sacrificing accuracy [11]. Song et al. introduced a weighting module and residual module into EDRNet, effectively filtering background noise and exhibiting strong robustness [21]. Feng et al. [22] introduced FcaNet and the Convolutional Block Attention Module (CBAM) based on ResNet50, achieving an accuracy of approximately 94%. However, the introduction of new modules brought about the issue of category imbalance. Although SSD is similar to YOLO in terms of speed and Faster R-CNN in terms of accuracy, SSD is less robust and requires higher debugging ability from the user. SSD adopts the idea of pyramidal feature hierarchy, but there is still the problem of insufficient feature extraction for small goals.

A lightweight algorithm is an algorithm designed for a specific scenario; they are usually simpler and faster. In order to facilitate the smooth execution of deep neural networks on mobile devices, researchers developed the MobileNet network model, which provides a trade-off between latency and accuracy [23]. Feng et al. [24] improved the accuracy of the MobileNet network by 3.18% on a strip steel surface defect dataset through the use of a data augmentation method. Lin et al. [25] combined the stochastic offline data enhancement algorithm, inverse residuals, and MobileNetV3 to enhance the model’s representation capability and generalization performance while maintaining a lower computational cost. Although the MobileNet algorithm enables deep neural networks to run smoothly on mobile devices, it exhibits low stability in terms of image processing accuracy in real production environments. Thus, there are limitations to its applicability.

YOLO initially attracted a lot of attention because of its simple loss function and the speed of real-time detection. The YOLO model has been updated through many generations, and different combinations of optimization have been attempted in various aspects such as detection head and loss function. YOLOv5 is an update of YOLOv3, which has faster speed [26,27] and higher accuracy [28,29]. In this paper, the YOLOv5s model was tested on the VOC2007 dataset, comparing its various parameters with other methods. Table 1 shows that YOLOv5s has better parameters in terms of Params (M), model size, GFLOPs, and FPS compared to other methods. Although YoloX-s has higher accuracy than YOLOv5s, it suffers from a significant decrease in FPS, which is not conducive to real-time monitoring of surface defects on steel strips. YOLOv7 achieves the highest mAP@0.95, but its other model parameters are larger, making it difficult to deploy on workshop equipment for actual production of steel strips. Additionally, YOLOv7 requires the use of pre-trained models and may not perform well on unfamiliar images such as steel strip pictures, making it challenging to make quick adjustments based on the actual situation. While YOLOv7-tiny has excellent parameters in various aspects, it and YOLOv7 both suffer from overestimated accuracy [30]. For example, if YOLOv7 is simulated for actual detection, setting the pre-processing size to a fixed 640 pixels would decrease mAP by 0.5%. Reducing the maximum number of predicted boxes and detection boxes from 30,000 to 1000 would decrease recall and mAP by 0.8%. Disabling one box to assign two categories would decrease mAP by 0.2%. We know that YOLOv7 is a modified version of YOLOv5, and the modular design of YOLOv5 allows for different adjustments based on specific situations. In comparison, YOLOv5s offers better and more convenient scalability.

A comprehensive analysis of the development of YOLOv1 to YOLOv8 is presented by Terven, J.R. et al. [31] The authors conclude that from YOLOv5 onwards, all official YOLO models have been fine-tuned with respect to the trade-off between speed and accuracy, with the aim of better adapting to specific applications and hardware requirements. YOLOv5 to YOLOv8 accuracies are not very different, while YOLOv5 has higher scalability and community resources compared to YOLOv8. When using YOLOv8 to test the NEU-DET dataset, we found that YOLOv8’s detection results for small targets are not satisfactory, and the training time will be longer compared to YOLOv5. Therefore, we finally chose to use YOLOv5 instead of YOLOv8.

Therefore, we chose YOLOv5 for strip surface defect detection and propose YOLOv5s-FPD. In order to meet the high requirements of small target detection and industrial inspection, we needed to strengthen YOLOv5 in terms of speed, number of model parameters and feature extraction capability. In terms of speed and number of model parameters, we chose DCNv2 (Deformable ConvNets v2) to reduce the amount of computation in the convolutional layer. In terms of feature extraction capability, we designed the CSBL multi-scale feature module for better feature fusion, ASFF and CARAFE to improve the sensitivity of small target feature extraction, and CBAM to adjust and assign the weights of features. Compared with YOLOv5s, the accuracy of YOLOv5s-FPD is improved by 2.6%. In order to be able to better improve the accuracy and robustness of small target detection, we designed the SSIN data enhancement method and allowed YOLOv5s-FPD to achieve 97.5% accuracy on the NEU-DET dataset.

### 2.2. Steel Surface Defect Data Set

Currently, there is no comprehensive and unified dataset available for detecting surface defects in steel products. Song et al. [32] proposed a NEU-CLS dataset consisting of six sample images. However, there is significant variation in the appearance of defects within the dataset. Furthermore, defect images are subject to variations in both illumination and material. A dataset of steel plate surface defects was created by PAO Severstal (Russia, Vologda Region) [33], containing 18,000 images for target detection. The lack of reference images created a significant challenge in enhancing these datasets. Matthias [34] addressed this issue by synthetically creating steel surface defect images, resulting in ten datasets. Each dataset included 1000 (2000) “defect-free” images and 150 (300) “defective” images in grayscale 8-bit PNG format. Tabernik et al. [35] proposed a dataset of steel surface defects based on real-world examples collected in controlled industrial settings. The reference dataset included 52 images with evident defects and 347 images without any defects, thus partially solving the issue of a shortage of reference images and facilitating the development of images of defects on steel surfaces.

## 3. Materials and Methods

### 3.1. Image Acquisition

#### 3.1.1. Description of the Data Set

The experiments in this research use NEU-DET, an open dataset of steel strip surface defects from Northeastern University, and the publicly available VOC2007 dataset. Since its release in 2022, the NEU-DET dataset has garnered significant attention among researchers due to its reliable image annotations, prevalent annotation categories, and a manageable count of 1800 images. Furthermore, its accessibility and user-friendly nature make it particularly appealing. On the other hand, the VOC2007 dataset, chosen for our extended experiments, mirrors the NEU dataset in terms of image size. Its reduced image count per class aligns with scenarios involving fewer samples. This characteristic positions VOC2007 as an ideal dataset for enhancing the model’s proficiency in detecting limited samples, while also serving as a benchmark for assessing the model’s robustness and generalizability.

**Figure 1:**
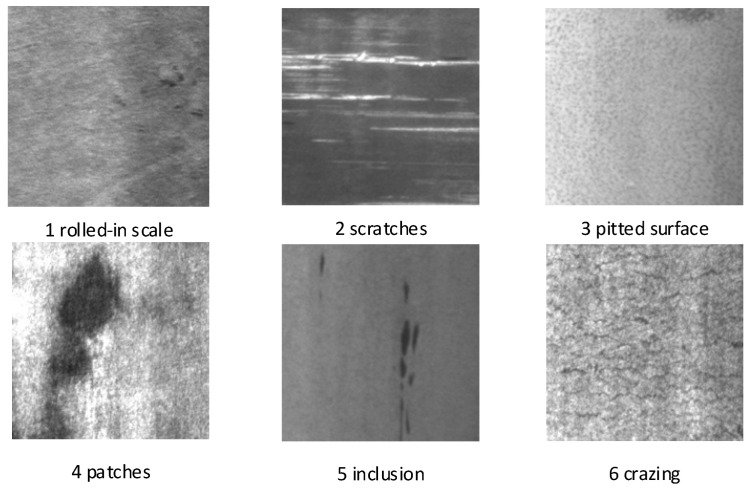
NEU surface defect dataset.

NEU-DET consists of six categories of hot-rolled strip surface defects (cracks or crazing, inclusions, pitted surfaces, patches, rolled-in iron oxide, and scratches)—as depicted in Figure 1—that account for the most frequent defects in hot-rolled strip surfaces.

The total dataset comprises 1800 images with 300 images per category, and each image has a size of 200 × 200 pixels. To increase the size of the dataset, image preprocessing is employed using seven methods, resulting in 18,000 images. The dataset is randomly partitioned into training, testing and validation sets, with an 8:1:1 ratio. The NEU dataset is characterized by the following features: (1) an equal number of instances for each class, with distinct feature differences, and (2) differing light biases and complex textured surface due to the unstable temperature of the production environment, making it a robust dataset for metal surface defects. These features render the NEU dataset to have common characteristics of metal surface defects found in industrial environments.

**Figure 2:**
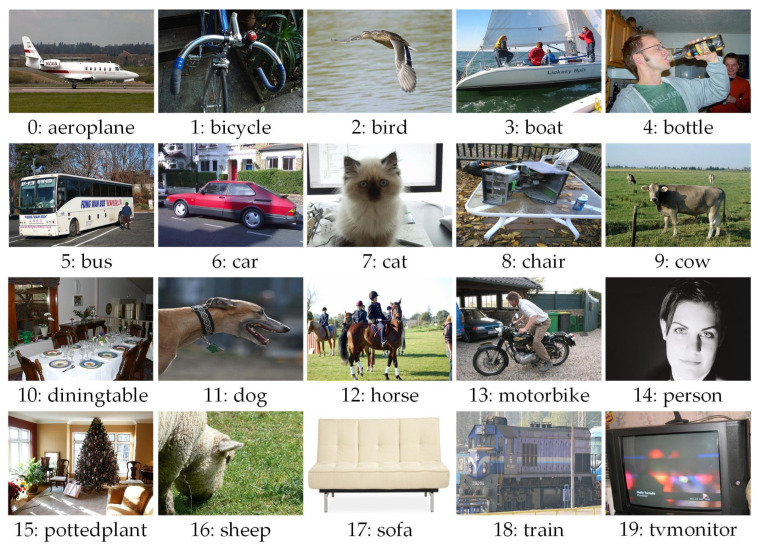
VOC2007 public dataset.

The public VOC2007 dataset consists of 20 classes, containing a total of 9950 images (as presented in Figure 2), with a resolution of approximately 500 × 350. These classes include aeroplane, bicycle, bird, boat, bottle, bus, car, cat, chair, cow, dining table, dog, horse, motorbike, person, potted plant, sheep, sofa, train, and TV/monitor. The experiment randomly generates the training, test, and validation sets in a ratio of 7:2:1.

#### 3.1.2. SSIE (Steel Strip Image Enhancement)

**Figure 3:**
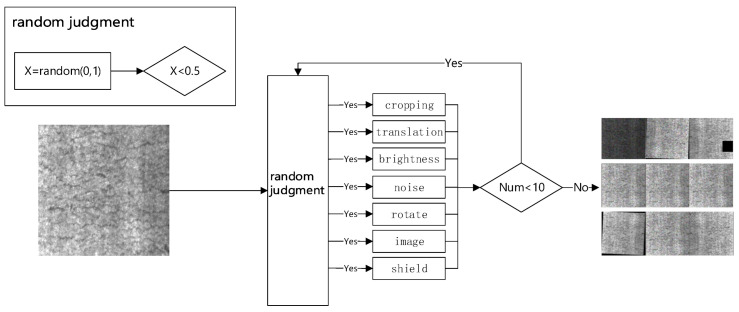
SSIE Module.

The strip steel processing process involves several procedures like cutting, galvanizing, and coating, among others, that may occur in a high-temperature, high-pressure, high-speed environment. Hence, the captured image is likely to contain noise, uneven lighting, and other factors that can affect the feature learning and judgement of convolutional network models. Thus, to ensure proper processing by the model, the input image needs to be adjusted on some parameters, tailored to the type of image that the model is good at processing. To underscore the image’s defect characteristics, this paper proposes an optional and efficient preprocessing method for strip steel surface images. The approach notably enhances the precision of the model for extracting features from images, as illustrated in Figure 3.

After inputting the image into the SSIE module, the image enters the random judgement step, where the processing method of the image is judged by the random parameter X ∈ (0, 1). All processing methods will perform a random judgement; X_cropping_ is the judgement value of the cropping method, X_translation_ is the judgement value of the translation method, and so on. When only X_cropping_ is less than 0.5, then the image only cropping processing method is performed to output the image. When only X_cropping_ and X_translation_ are less than 0.5, then the image is first processed by the cropping method, and then the cropping processed image is then processed by the translation method to output the image. The Num parameter is incremented by 1 every time a picture is output, and the loop ends when Num is greater than or equal to 10.

#### 3.1.3. Algorithm Description

The small color difference between the target defects and the background in the strip surface defect data image makes it difficult to define the prediction frame boundary of the target, leading to misdetection and omission. Thus, constructing a multilevel sensory field that enables the model to localize a more comprehensive target will increase the weighting of target defects.

**Figure 4:**
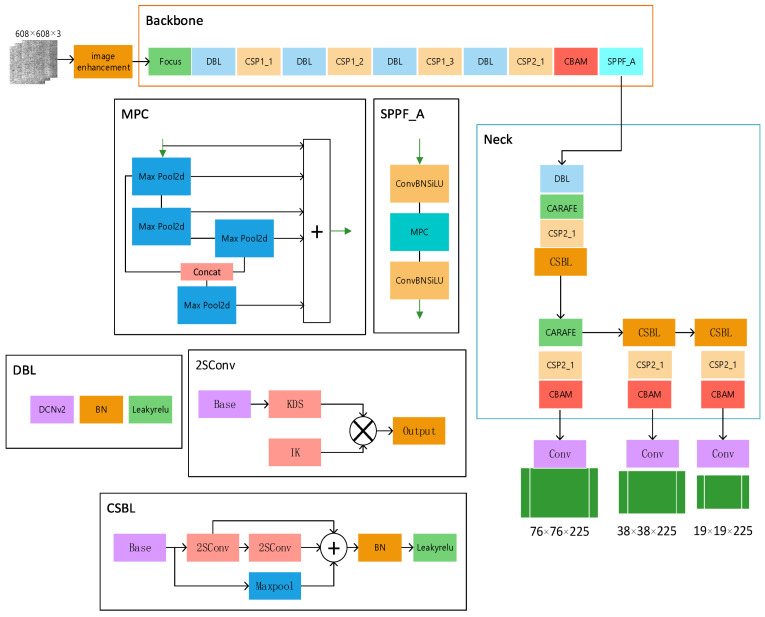
YOLOv5s-FPD network structure.

Using this information, we constructed the YOLOv5s-FPD model structure depicted in Figure 4. The input image resolution can be based on the general 640 resolution or the image resolution of the dataset. We hope that the model can cope with the complex situation of actual production, so we choose the general 640 resolution as the base point for testing, and the accuracy of 608 resolution is higher in the results, so we set the input image resolution to 608. To enhance the model’s ability to match the dataset, we added the CBAM attention mechanism to improve its fine-grained feature representation. To enhance the image’s recognizability, we preprocessed it by performing operations such as changing its histogram settings, brightness, and adding noise, among others, to improve its recognition ability. Since the model will be used in real-life production, it is crucial to improve its accuracy while keeping its size and speed maximized. To achieve this, we replaced the upsampling with CARAFE, applied ASFF as the detection head, and used DCNv2 convolution to enhance the model’s ability to adapt to the image’s fine features. To reduce the computational effort caused by the extra convolution, we changed the C3 bottleneck layer in the neck part of the model to CSBL. To overcome the limitations in feature detection caused by the field-of-view pooling and splicing, we introduced SPPF_A, and innovatively replaced the last layer of concatenation in the head part of the original model with BiFPN.

**Figure 5:**
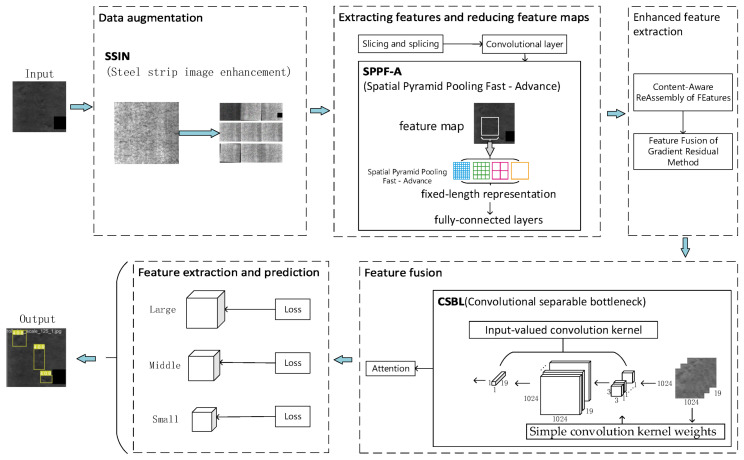
YOLOv5s-FPD Concept Map.

In order to describe the role of each module more clearly, a conceptual diagram of YOLOv5s-FPD has been drawn. As shown in Figure 5, the SSIN (steel strip image enhancement) module performs data enhancement on the image after inputting the image. The data-enhanced image is processed using the convolutional layer with the C3 module and gradient fusion and adaptive output scaling of features in the SPPF-A (Spatial Pyramid Pooling Fast-Advance) module. After inputting the multi-scale feature map into the upsampling and upper layer convolutional feature gradient fusion, the CSBL (Convolutional Separable Bottleneck) module is used to increase the depth and sensory field of the network to improve the ability of feature extraction; finally, the features are analyzed and extracted and predicted.

### 3.2. Improvement Methods

#### 3.2.1. Evolutionary Separable Bottleneck (CSBL) Development

The efficacy of convolutional neural networks (CNNs) for image recognition has been established in prior models and numerous studies. Since the development of AlexNet, deep CNNs have demonstrated high accuracy in target recognition. Deep convolution yields high accuracy rates, but also results in millions of parameters, even with GPUS, which require substantial computing space. Hence, improving convolution efficiency and computation time have become the new areas of focus. Convolutional neural networks like VGG take up more than 80% of the runtime and space, implying that to enhance performance, more convolutional layers are needed while decreasing the computation in each individual convolutional layer. Therefore, we propose the CSBL module with the aim of decreasing computation while preserving high accuracy levels.

**Figure 6:**
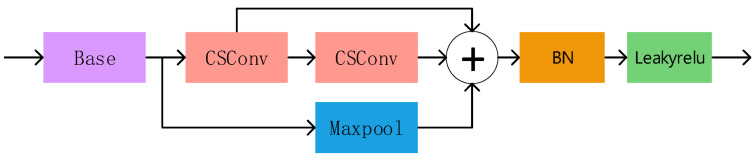
CSBL module. Where “+” is for splicing.

Replacing the convolution with the Simple Separable Convolution, the CSBL module depicted in Figure 6 reduces the computation amount and speeds up model computation. Moreover, when combined with the spatial pyramid model, the fusion of multi-scale feature structure becomes better suited to the dataset picture, accommodating for diverse defective features of the variable sensory field.

The separable convolution part of 2SConv reduces the computational effort from the convolution kernel-channel correspondence, assuming that the size of the input image a is 0utput=height×weight×channel, The size of a single convolutional kernel is v=s×s×channel, *stride* = 2, *filters* = 64, *pad* = 1, then the output is shown in Equation (1):

(1) $$h=1+\left(height+2\times pad-s\right)/stride$$

$$w=1+\left(weight+2\times pad-s\right)/stride$$

Output=h×w×filters

where *pad* = 1 is meant as a judgement condition, *pad* = 1, i.e., *pad* is true, *pad* = s/2. If *pad* = 0 i.e., *pad* is false, *pad* = 0.

Next, compare where the difference between ordinary convolution and 2SConv lies. Again, taking image a as the object of analysis, the computation of a single convolution kernel for ordinary convolution can be obtained as shown in Equation (2):

(2) $$D_{ck}=height\times weight\times s\times s\times channel$$

if the total number of convolution kernels is *N*, the total computation is $W_{ck}=D_{ck}\times N$.

For 2SConv, only one convolution kernel is used for each channel, and the output result is *M* feature maps with channel 1. The second step combines the *N* feature maps with convolution kernels of size 1 × 1 × *M* to get an output feature map with channel *N*, and M is the number of channels in the previous layer. Thus, 2SConv first step Depthwise Convolution computation is shown in Equation (3):

(3) $$D_{ck}=height\times weight\times s\times s\times M$$

the second step Pointwise Convolution operation is similar to the regular convolution operation, the size of the convolution kernel is 1 × 1 × *M*, there are several convolution kernels have several output feature maps, and the amount of computation is shown in Equation (4):

(4) $$P_{ck}=\mathrm{height}\times \mathrm{weight}\times M\times N$$

the total 2SConv calculation is shown in Equation (5):

(5) $$W_{ck}=D_{ck}+P_{ck}$$

**Figure 7:**
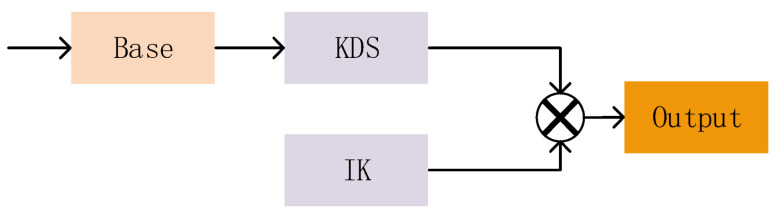
2SConv convolution. Where “×” means product.

The convolution operation of 2SConv is implemented through two distinct components, as illustrated in Figure 7, namely the input-valued convolution kernel IK and the deep convolution kernel weights (KDS).

1. Input-valued convolution kernel (IK)

To perform a simple convolution operation for 2SConv, we define the convolution kernel based on the input with the same width and height as the original convolution tensor. Then, we carry out an integer shaping transformation. The shaping computation discards nearby fine-grained features, but it brings forth distinct advantages. Firstly, defining a similar kernel enhances the clarity of feature edges. Also, various modules are integrated into the model to expand the field of view and fuse different features. Even when some features are identified as invalid, they still require the same computation. Secondly, a model with an attention mechanism evaluates the importance of each image feature based on its relevance. Combining the above two points, this shaping computation causes minimal loss of valid features while greatly reducing the computation requirements compared to floating point computation.

2. Simple convolution kernel weights (KDS)

IK is a light plastic component of CSBL that effectively speeds up operations and reduces memory load. In order to reduce the unwanted impact that using IK might have on the results and to enhance the balance of the method in terms of efficiency and accuracy, we chose to add the KDS module. This module reads the spatial and channel weights of the input feature parameters, while utilizing minimal parametric quantities. Firstly, the input features are multiplied by IK. Achieving the effect of increasing the speed of computation requires advanced integer computation techniques. Next, the obtained results are multiplied by KDS. The weights are calculated for each feature before the results are output, achieving a similar effect to that of normal convolution. To better integrate with the other modules of the model, we introduced the spatial pyramid structure in KDS. This structure scales the field of view by fusing features at three scales for 2SConv and Maxpool.

#### 3.2.2. SPPF_A Design

Spatial Pyramid Pooling (SPP) is a pooling method used in computer vision, which has been improved through SPPF and is known to deliver the same output at a faster pace. Spatial pyramid pooling can efficiently rescale feature maps that have varying scales to create uniform scales. However, as per the source code of YOLOv5, SPP and SPPF utilize the same pooling method by padding. As a result, the size of the feature maps remains unchanged before and after pooling. The primary role of SPPF in YOLOv5 is to merge features of multiple scales within the same feature map. SPPF concatenates features of varying scales within the same feature map to enhance performance.

**Figure 8:**
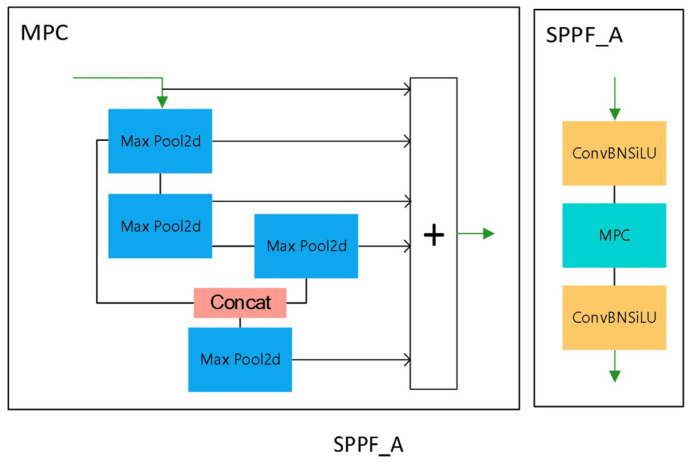
SPPF_A module. Where “+” is for splicing.

Serial computation of four MaxPools with kernel of 5 × 5 is performed by SPPF_A, as shown in Figure 8. Our proposition is that SPPF_A combines the concatenation of the first MaxPool and the third MaxPool to focus more on large and fine targets. The reason for this is that the resolution of the strip surface defect image is low, the defects are small, and their range is not fixed. When there are only a few defects, the labelled area is minimal, whereas when there are many defects, the labelled area covers almost the entire image. Therefore, the model needs to focus more on the domain of the variable field of view. The spatial pyramid structure of SPPF_A is compatible with the output of different picture scales and sizes, and it extracts features corresponding to the appropriate scale according to the image’s different scales. Extracting features based on the different scales of the image and combining them can lead to improved recognition accuracy.

### 3.3. Design of the Loss Function

The loss function used in this paper consists of three aspects: rectangular box, confidence and classification probability, and image extraction is performed using mask mask. We divide the whole image into N × N grids with different sizes of field of view, and judge which grid the prediction box is in using mask mask. The formula for determining whether to keep the prediction box is shown in Equation (6):

(6) $$t_{w0}=\max\left(\frac{w_{gt}}{w_{0}},\frac{w_{0}}{w_{gt}}\right);\;t_{h0}=\max\left(\frac{h_{gt}}{h_{0}},\frac{h_{0}}{h_{gt}}\right)$$

where *w_0_* and *h_0_* are the width and height of the grid and *w_gt_* and *h_gt_* are the width and height of the prediction box. Define a threshold value *x*. When *t_w0_* and *t_h0_* are greater than *x*, they are retained; otherwise, this mesh is excluded from the calculation of rectangular box loss and classification loss.

Define the loss function as: Loss=a×lossobj+b×lossrect+c×lossclc, where *lossrect* is the rectangular box *loss*, *lossobj* is the confidence *loss*, and *lossclc* is the classification *loss*. *a*, *b*, and *c* are the weights, which serve to adjust for the target features in different field of view.

#### 3.3.1. Matrix Frame Loss

In target detection, the most commonly used evaluation criterion is IoU. IoU represents the intersection and fusion ratio between the target frame and the detection frame, a larger ratio indicates more accuracy, with a scale-insensitive property, according to the current value of IoU, which can reflect the direct effect of the predicted value and the *gt* value. The formula of the IoU loss function is shown in Equation (7), which represents the difference in the intersection and fusion ratios between the predicted frame A and the real frame B. The IoU loss function can be calculated by using the IoU loss function:

(7) $$L_{\mathrm{IoU}}=1-\mathrm{IoU}\left(A,B\right)$$

However, the shortcoming of IoU is that it can only compare the difference between them and cannot reflect the shape of the two when they overlap, as well as the size of the overlap.

GIoU adds a minimum envelope parameter C on top of IoU, and the formula is shown in Equation (8):

(8) $$L_{\mathrm{GIoU}}=1-\mathrm{IoU}\left(A,B\right)+\left|C-A\cup B\right|/\left|C\right|$$

The minimum enclosing frame C calculates the size of the overlap between A and B. However, if A and B are not initially in contact, the GIoU consumes a lot of time in moving the prediction frame A, which affects the speed of convergence of the loss.

We use CloU as the loss for determining the bounding box, compared to the earlier IoU and GIoU losses adding the diagonal distance, the distance between the center points of the two frames, and the aspect ratio of the two frames as shown in Equation (9):

(9) $$L_{\mathrm{CIoU}}=1-\mathrm{IoU}+\frac{\rho^{2}\left(b,b^{gt}\right)}{c^{2}}+\alpha v$$

where $\rho^{2}\left(b,b^{gt}\right)$ is the Euclidean distance between the centroids of the predicted and real boxes, and c is the diagonal distance that contains the smallest closed region of the predicted and real boxes. Where v is used to measure the consistency of the relative proportions of the two rectangular boxes, and α is the weighting coefficient as shown in Equation (10):

(10) $$v=\frac{4}{\pi^{2}}{\left(arctan\frac{w^{gt}}{h^{gt}}-arctan\frac{w}{h}\right)}^{2};\alpha =\frac{v}{\left(1-\mathrm{IoU}\right)+v}$$

#### 3.3.2. Loss of Confidence and Loss of Categorical Probability

The computational complexity is reduced by replacing the softmax function with binary cross entropy for each label to calculate the likelihood of inputting a specific label when computing the classification loss for training. The binary cross entropy formula is shown in Equation (11):

(11) $$\;Loss=-\frac{1}{N}\sum_{I=1}^{N}y_{i}\cdot \log\left(p\left(y_{i}\right)\right)+\left(1-y_{i}\right)\cdot \log\left(1-p\left(y_{i}\right)\right)$$

where *y* is the binary label, $y\in \left[0,1\right]$, *p(y)* is the probability that the output belongs to the label *y*. Binary cross entropy is used to judge how good or bad a binary model’s predictions are; for a label *y* of 1, if the predicted value *p(y)* tends to 1, then the value of the loss function should tend to 0, and vice versa for a label *y* of 0.

### 3.4. Selection of Backbone Network

The role of the backbone network is to extract features and convert the original strip defect images into multi-layer feature maps. Our model backbone network mainly consists of Focus, DCNv2, C3 and SPPF_A modules.

The focus module is similar to downsampling, with the way of slicing the high-resolution stripe image sampling into four low-resolution stripe images, but at the same time retains all the information of the image. The size of 3 640 × 640 images was incorporated into the focus module after extracting a value over the columns; the result is 4 320 × 320 strip images, splicing after the channel from the RGB three-channel into a 12-channel. The role of the focus module is to merge the calculation of multiple convolutional layers to reduce the amount of computation and the number of parameter roles.

DCNv2 consists of a variability convolution layer, a BN layer and an activation function. The variability convolution adds an offset without changing the ordinary convolution operation, and 2N convolution kernels are convolved with the feature map to obtain the parameters of *x* and *y* directions for each position offset, which are added with the ordinary convolution result to obtain the final result as shown in Equation (12):

(12) $$y\left(p\right)=\sum_{k=1}^{k}w_{k}\cdot x\left(p+p_{k}+\Delta p_{k}\right)\cdot \Delta m_{k}$$

where $\Delta p_{k}$ is the offset, $\Delta m_{k}$ is the sampling point weight, and $\sum_{k=1}^{K}w_{k}\cdot x\left(p+p_{k}\right)$ and is the position of each value of the ordinary convolution.

The C3 module consists of one convolutional block with a stride of 2 and two convolutional blocks with a stride of 1. The feature map is first passed through the convolutional block with a stride of 2 to increase the receptive field and reduce the computational effort while reducing the size. Next, the channel is increased by two convolutional blocks with a stride of 1 to better preserve the local features of the object.

The SPPF_A module places different sizes of receptive fields on the same feature map to capture different scales of feature information. There are four maxpool layers in the SPPF_A module. To realize the ability to capture features at different scales, we store the output of each maxpool layer that the feature map passes through and then concatenate the outputs. In the case of stripe defect images with high noise and uneven light, we concatenate the outputs of the first and third maxpool layers as the input of the last maxpool layer, which can be better adapted to the stripe defect features.

## 4. Results

The hardware and software environment used in the experiments of this paper are as follows: the CPU is an Intel(R)i5-12400(Intel, Santa Clara, CA, USA), GPU is an NVIDIA RTX3060(NVIDIA, Santa Clara, CA, USA), operating system is win10, Python3.7.6 is used, and the deep learning framework is selected as PyTorch1.6.0. The YOLOv5s model is used as the benchmark for this experiment, and during the training process, the batch size is set to 32, and the training epoch is set to 400.

### 4.1. YOLOV5s-FPD Model Experiment

In order to verify the improvement of the YOLOv5s FPD model in target detection results, several classical algorithms for strip steel surface defect detection are selected, including Faster-RCNN, SSD, YOLOv3, YOLOv5s, and YOLOv5m, several classical target detection algorithms. Before running the experiments, the NEU-DET dataset is pre-processed. The pre-processed dataset is given to each model for experimentation. The final experimental results are shown in the table, including the average mAP of all classes as well as the individual AP accuracies.

From Table 2, it can be seen that adding convolution to the original YOLOv5 model does not improve the detection accuracy because it cannot locate the defective features in the image. The YOLOv5s-FPD model outperforms the original YOLOv5-s and other classical algorithms in terms of comprehensive detection performance as well as the six defect categories, indicating that the CBAM attention and the ASFF detector head have an improved effect in detecting defects on the steel surface. The FPS of YOLOv5s-FPD is 13% lower than that of YOLOv5-s and 45% higher than that of YOLOv5-m. YOLOv5-m has a larger model structure and higher computational complexity, but increasing the depth of convolution and the number of channels does not improve the accuracy.YOLOv5s-FPD, in order to deal with the characteristics of the steel image, which has insignificant color difference and the defects are small and dispersed, chooses to add convolutional layers, and upsampling, pooling, and other steps were carried out to achieve the expanded field of view domain by splicing the results at different scales. The results show that these ideas are effective and that accuracy improvement can indeed be achieved by performing different-scale splicing while trying to maintain the processing speed.

From Table 3, it can be seen that on the VOC2007 public dataset, YOLOv5s-FPD-c has a 4.6% improvement in mAP50 compared to YOLOv5-s. After adding variable field of view domains to multiple modules of the model, it is able to enrich multi-scale information and realize multi-scale feature fusion when dealing with complex image information, and it can better complete the image detection task. Meanwhile, the above data also illustrate that the CSBL module’s method of combining depth-separable convolution and spatial pyramid structure has a certain degree of generalization and can cope with different types of datasets.

### 4.2. Ablation Experiment

To verify that the improved part can improve model performance, this section performs ablation experiments on the NEU dataset. In ablation experiment 1, one of the improved modules is removed one at a time and the improved structure is added back to the original YOLOv5 module. By comparing the mAP and FPS results, the effect of different structures on the results is tested. The experimental dataset still uses the NEU-DET dataset.

As shown in Table 4 and Table 5, when CBAM is not used, the average accuracy mAP50 decreases by about 1.7%, indicating that the attention module helps to extract more accurate feature information from defect images with complex backgrounds; when the ASFF adaptive feature fusion module is not used, the average accuracy mAP50 decreases by about 0.9%, indicating that making full use of the semantic information of the high-level features and the fine-grained features of the underlying features is a strip steel defect detection; when CARAFE is not used, the average accuracy mAP50 decreases by about 0.8%, indicating that upsampling with a large receptive field can better ignore the background to extract features; when SPPF_A is not used, the average accuracy mAP50 decreases by about 0.6%, indicating that the feature extraction has a large impact on the recognition; when BiFPN is not used, the average accuracy mAP50 decreases by about 0.1%, indicating that band-weighted feature fusion has a positive effect to some extent; when CSBL is not used, the FPS decreases from 138 to 103, indicating that separable convolution can significantly improve the computational efficiency, and 2SConv replaces a part of floating-point operations with integer computation, which improves the efficiency under the premise of ensuring that the number of computations remains unchanged, whereas spatial pyramid mode makes it possible to achieve lightweighting while maintaining a similar effect of ordinary convolution.

Ablation experiments 1 and 2 illustrate the validity of the improved model.

### 4.3. Comparative Analysis of Different Modules

#### 4.3.1. Data Enhancement Comparison

As can be seen from Table 6 and Table 7, the data enhancement in this paper improves the model performance significantly. The data are processed in seven ways: cropping, panning, changing brightness, adding noise, rotating angle, mirroring, and cutout, and each image is increased by 9 processed images each, and the total number of images in the dataset is increased from 1800 to 18,000. After preprocessing, the mAP index of the original YOLOv5 model increased from 57.8% to 95.7%, while the mAP index of the YOLOv5s-FPD model increased from 60.4% to 97.5%, in which the detection accuracy of the model for each defect category was effectively improved after image preprocessing, especially the accuracy of the indusion category, which increased by 50%. The experimental results show that the preprocessing method can bring effective improvement to the defect detection performance.

**Figure 9:**
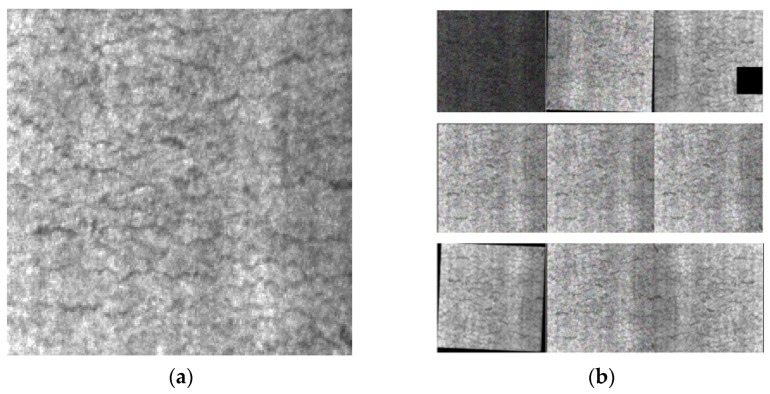
Data enhancement (**a**) cracked (Cr) label original figure; (**b**) data enhancement effect diagram.

Figure 9 shows the processing effect of one of the cracked (Crazing, Cr) labelled images in the dataset; through observation, it can be seen that the data-enhanced image has a large gap with the original image and the robustness of the model is improved.

#### 4.3.2. Comparison of Forecast Frames

**Figure 10:**
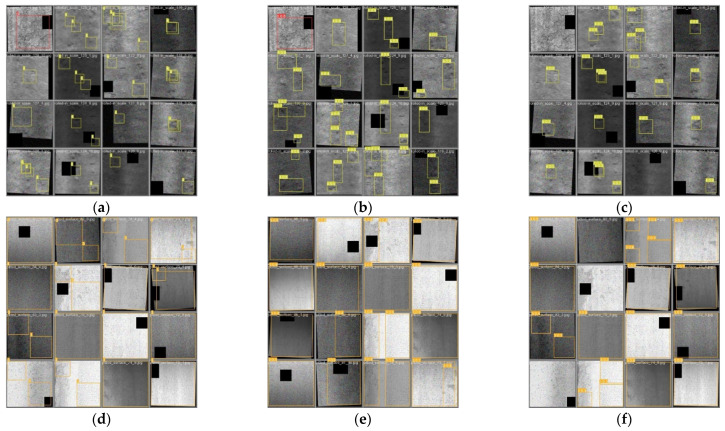
Comparison of detection frames. (**a**,**d**) Labeling of original; (**b**,**e**) YOLOv5s-PFD; (**c**,**f**) YOLOv5s.

As can be seen from Figure 10, YOLOv5s-FPD is able to make better judgments when dealing with features that are similar to the background and can also detect and correctly classify overlapping defects in the image. On the other hand, YOLOv5s misses more overlapping features and is more influenced by the background when detecting larger features. It can be seen that the traditional YOLOv5s is generally effective in detecting large targets and fine-grained small targets, SPPF is not able to retain all the features in the image, and some of the images cannot be detected and classified. SPPF_A can eliminate the interference of the background in the image by varying the spatial pyramid structure and make use of the different field of view to extract complex features. The results show that both the classification accuracy and the IOU value have been improved, and SPPF_A is able to better identify the features in the strip image.

#### 4.3.3. CSBL Module Convolution Comparison

From Table 8, we can see that CSBL is reduced in all four aspects of layers, parameters, FPS and GFLOPs, the number of parameters of GFLOPs is reduced by 14%, the number of images processed per second (FPS) is improved by 34%, and the accuracy mAP50 (%) can be maintained, which shows that the changes are effective.

## 5. Discussion

The defects on the surface of steel strip have random characteristics, and even within the same category, there can be significant differences in shape and size. Due to the high temperature and dust in the workshop, the images captured in the production environment do not have high clarity, which poses a challenge to defect detection and classification. In this study, separate data statistics were performed on six types of defect images in the NEU strip steel dataset. The experimental results showed that YOLOv5s FPD outperformed the baseline in detecting six types of defects. Another challenge is the influence of background noise and light on the detection of surface defects on steel strip. The use of weights during upsampling and downsampling helps to ignore the influence of background. In addition, the use of image enhancement can effectively compensate for the influence of the data set on the accuracy of the model. In this study, a highly versatile image enhancement method was developed and applied to the NEU dataset. The experimental results showed that this method can improve the accuracy by about 37%. Future research should focus on optimizing image processing techniques to further reduce the impact of light changes and improve detection accuracy.

In addition, when conducting the experiments, we found that the data results of YOLOv5-m are not satisfactory, as shown in Table 9, and the width and depth of YOLOv5-m are larger than that of YOLOv5-s, but the result is instead that the latter has a higher accuracy rate. Comparing the AP (%) values in the table, we find that the accuracy of YOLOv5-m is reduced in each type of image, which indicates that on strip defect detection, it may not be very effective in increasing the width and depth of the YOLO model.

**Figure 11:**
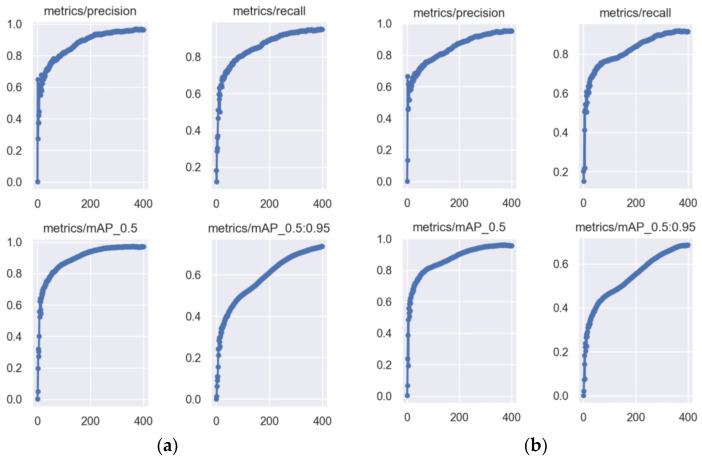
(**a**) YOLOv5s; (**b**) YOLOv5-m. The blue dot represents the accuracy of the current epoch.

We compare the parameter plots of the model training, as shown in Figure 11, with the training parameters of YOLOv5s on the left and YOLOv5-m on the right. The training was performed for a total of 400 epochs and used data augmentation, with the baseline being YOLOv5s. We can see from the comparison in the graphs that the curve for YOLOv5s is more stable, the precision and recall rise faster, and the mAP@0.5 parameter is close to its maximum value at around the 300th epoch. On the other hand, YOLOv5-m reaches the maximum value of mAP@0.5 only at around 370 epochs, which suggests that YOLOv5-m needs more epochs to maximize the performance of the model.

**Figure 12:**
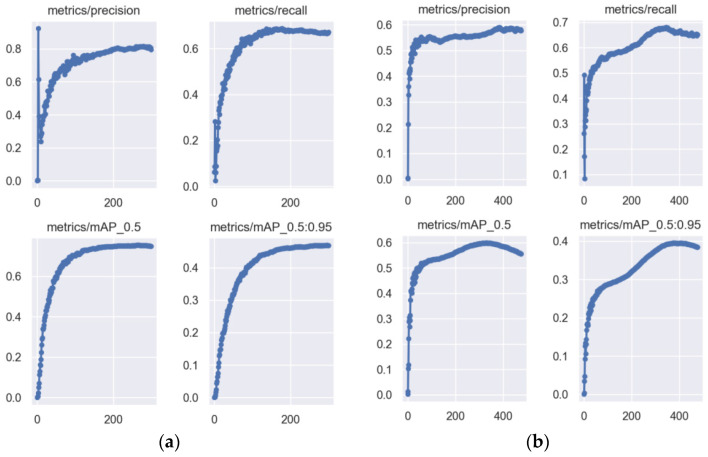
(**a**) YOLOv5s-FPD; (**b**) YOLOv5-m. The blue dot represents the accuracy of the current epoch.

To achieve the best performance of YOLOv5-m on the NEU dataset, we conducted a YOLOv5-m verification experiment. We did not use data enhancement for 500 epochs in this experiment, and the baseline was YOLOv5s FPD. Figure 12 shows that the YOLOv5s FPD curve on the left is stable and near the highest mAP@0.5 value around the 200th epoch, which indicates the model’s ability to learn the features in the image. YOLOv5m on the right approached the highest mAP@0.5 value at around the 300th epoch and then started to decline, indicating that the model had fulfilled its potential. Upon comparing the curves on the left and right sides, we observed that YOLOv5m did not perform well, and increasing the model’s width and depth led to a longer training time. Thus, this proves that YOLOv5s FPD is more versatile and has performed better in the challenge posed by the small dataset samples.

As seen from the above discussion, the integration of the YOLOv5s-FPD model into industrial equipment has great potential in advancing industrial automation and intelligence. Since the motion, vibration and noise of the equipment may affect the detection accuracy and stability of the model, the scalability and adaptability of the model will become more important in the actual industrial production environment. Integrating YOLOv5s-FPD models into industrial equipment requires comprehensive consideration, extensive testing and optimization to develop viable application solutions.

## 6. Conclusions

The rapid inspection and detection of surface defects on strip steel is of great significance for strip steel production. In this paper, we apply the combination of efficient convolution and separable convolution to the defect detection of strip steel by proposing the CSBL module and investigating its structure. Secondly, in order to make the model prediction frame better applied to strip steel defect detection, we propose the SPPF_A module to make the spatial pyramid structure more suitable. Since ASFF is compatible with the spatial pyramid structure, we choose ASFF for feature fusion. Finally, to enrich the information of the dataset, we propose a data enhancement method. The YOLOv5-FPD model proposed in this study has the following main outstanding contributions:

(1) This paper proposes a deep learning algorithm suitable for strip surface defect detection, and analyzes the structural features of YOLOv5 network and some problems in strip surface defect detection;
(2) In this paper, Faster RCNN, SSD, YOLOv3, YOLOv5-s, YOLOv5-m, and YOLOv5s-FPD are compared and analyzed for GFLOPs, detection speed FPS, mAP@0.5, and AP accuracy for each kind of defect, among which YOLOv5s-FPD has the highest detection speed and accuracy.
(3) In this study, YOLOv5s is improved by proposing SPPF_A fine-grained spatial pyramid pooling and constructing a CSBL multiscale feature module. The improved YOLOv5s-FPD has better recognition ability for strip defect images with strong background noise and strip defect images that are highly similar to the background.

The experimental results indicate that the present model is effective. However, we still need to make modifications in several places; for example, the model is difficult to accurately feature extract for noisy backgrounds, and the weights in the field of view domain are not well adjusted for specific images. This kind of image with difficult to distinguish backgrounds needs more to be more abundant for model training; however, data enhancement can only be carried out evenly for all images, so this situation needs to be proposed as a targeted solution. The next step is to maintain the accuracy of the model while further reducing the number of parameters of the model to speed up the detection.

## Figures and Tables

**Table 1:** Comparison of YOLO algorithms.

Method	Params(M)	GFLOPs	Model Size (M)	FPS	mAP@0.95
YOLOv5s	7.02	16	14.4	95	43.5
YOLOv3	61.53	193.8	120.5	54.6	38.5
YOLOv4	52.5	119.8	100.6	55	39.7
YOLOv5m	20.9	48.0	42.2	74	44.8
YOLOX-s	8.94	26.6	18.5	61	45
YOLOv6s	17.19	44.1	36.3	97	42
YOLO7	36.49	103.5	74.8	87	46.8
YOLO7-tiny	6.01	13.1	12.3	128	42.5

**Table 2:** Comparison experimental results of YOLOv5s-FPD on NEU-DET.

Arithmetic	Map@0.5	Map@0.95	AP (%)						FPS	GFLOPs
			Cr	In	Ps	Pa	Rs	Sc		
Faster RCNN	89.4	66.2	78.8	82.4	96.6	96.8	85.4	96.5	58	206.7
SSD	62.3	46.5	32.7	63.1	69.6	71,9	58.4	78.5	73	87.5
YOLOv3	88.8	65.7	76.4	82.6	93.7	94.5	84.4	96.5	116	193.8
YOLOv5-s	95.7	68.6	96.8	91.3	98.8	96.4	95.2	95.9	151	16
YOLOv5-m	95.3	65.3	96.1	91	98.4	96.1	94.8	95.5	93	48.3
YOLOv5s-FPD	97.5	73.7	98.7	94.6	99.5	97.8	96.8	97.6	138	20.2

**Table 3:** Comparison experimental results of YOLOv5s-FPD on VOC2007.

Arithmetic	mAP@0.5	mAP@0.95	Precision/%	Recall/%
Faster RCNN	66.4	38.2	68.3	61.7
SSD	60.2	33.2	61.7	56.8
YOLOv3	67	38.5	70.4	61.5
YOLOv5-s	70.7	43.5	72.9	64.6
YOLOv5s-FPD-a	71.4	44.9	75.6	64.1
YOLOv5s-FPD-b	72.6	45.5	74.9	67.2
YOLOv5s-FPD-c	75.3	46.9	81.5	67.6

**Table 4:** YOLOv5s-FPD ablation experiment 1.

Arithmetic	mAP@0.5	mAP@0.95	FPS	AP (%)					
				Cr	In	Ps	Pa	Rs	Sc
CBAM	95.8	69.2	144	97.4	91.7	98.2	96.2	95.4	96.1
ASFF	96.6	70.8	146	98.4	93.1	99.5	96.2	96.4	96.5
CARAFE	96.7	71.1	140	98.3	93.1	99.5	96.6	96.6	96.3
SPPF_A	96.9	72.3	126	99.1	93.7	99.5	97.1	96.7	95.2
DCNv2	97.4	73.4	131	98.6	94.5	99.5	97.8	96.4	97.6
CSBL	97.4	73.5	103	98.7	94.6	99.5	97.6	96.7	97.4
YOLOv5s-FPD	97.5	73.7	138	98.7	94.6	99.5	97.8	96.8	97.6

**Table 5:** YOLOv5s-FPD ablation experiment 2.

CBAM	ASFF	CARAFE	SPPF_A	DCNv2	CSBL	mAP50 (%)	mAP95(%)	FPS
✓	×	×	×	×	×	96.3	70.2	150
✓	✓	×	×	×	×	96.6	70.9	132
✓	✓	✓	×	×	×	96.8	71.0	129
✓	✓	✓	✓	×	×	97.1	72.3	117
✓	✓	✓	✓	✓	×	97.4	73.5	103
✓	✓	✓	✓	✓	✓	97.5	73.7	138

**Table 6:** YOLOv5s data enhancements.

Arithmetic	mAP@0.5	mAP@0.95	AP (%)					
			Cr	In	Ps	Pa	Rs	Sc
YOLOv5s	57.8	33.7	60.7	41	55.6	72.4	54.1	62.7
YOLOv5s + SSIE	95.7	68.6	96.8	91.3	98.8	96.4	95.2	95.9

**Table 7:** YOLOv5s-FPD data enhancements.

Arithmetic	mAP@0.5	mAP@0.95	AP (%)					
			Cr	In	Ps	Pa	Rs	Sc
YOLOv5-FPD	60.4	35.8	64.7	45.3	58.2	79.5	54.3	60.4
YOLOv5-FPD + SSIE	97.5	73.7	98.7	94.6	99.5	97.8	96.8	97.6

**Table 8:** CSBL convolution calculations.

	Layers	Parameters	FPS	GFLOPs	mAP50 (%)
ordinary convolution	214	7035811	103	16	97.4
CSBL	186	6009507	138	13.9	97.5

**Table 9:** Results of comparison experiments on NEU-DET.

Arithmetic	mAP@0.5	mAP@0.95	AP (%)						FPS	GFLOPs
			Cr	In	Ps	Pa	Rs	Sc		
YOLOv5-s	95.7	68.6	96.8	91.3	98.8	96.4	95.2	95.9	151	16
YOLOv5-m	95.3	65.3	96.1	91	98.4	96.1	94.8	95.5	93	48.3
YOLOv5s-FPD	97.5	73.7	98.7	94.6	99.5	97.8	96.8	97.6	138	20.2

## Data Availability

The data that support the findings of this study are available in [NEU-DET] and [VOC2007] at [http://faculty.neu.edu.cn/songkc/en/zhym/263264/list/index.htm] and [http://host.robots.ox.ac.uk/pascal/VOC/voc2007/index.html].
